# Increasing lay-people’s intentions to initiate CPR in out of hospital cardiac arrest: Results of a mixed-methods ‘before and after’ pilot study of a behavioural text-message intervention (BICeP)

**DOI:** 10.1016/j.resplu.2022.100312

**Published:** 2022-10-06

**Authors:** Barbara Farquharson, Eileen Calveley, Gareth Clegg, Brian Williams, Pam Ramsay, Lisa Macinnes, Claire Torrens, Diane Dixon

**Affiliations:** a NMAHP Research Unit, University of Stirling, FK9 4LA, United Kingdom; b University of Highlands and Islands, United Kingdom; c University of Edinburgh, United Kingdom; d University of Dundee, United Kingdom; e University of Stirling, United Kingdom; f University of Aberdeen, Scotland

**Keywords:** Lay-people, Out-of-hospital cardiac arrest, Initiation, Behaviour, Text-message

## Abstract

**Background:**

Prompt, effective cardio-pulmonary resuscitation (CPR) increases survival in out-of-hospital cardiac arrest. However, CPR is often not provided, even by people with training. Low confidence, perceptions of risks and high emotion can prevent initiation of CPR. Behaviour-change techniques may be helpful in increasing CPR rates.

**Aim:**

To pilot a text-message behavioural intervention designed to increase intentions to initiate CPR, explore participant responses and pilot methods for future randomised controlled trial of effectiveness.

**Methods:**

A ‘before and after’ pilot study plus qualitative interviews was undertaken. Participants were lay-people who had undertaken CPR training in previous 2 years.

Participants were sent an intervention, comprising 35 text-messages containing 14 behaviour-change techniques, to their mobile phone over 4–6 weeks.

*Primary outcome:* intentions to initiate CPR assessed in response to 4 different scenarios.

*Secondary outcomes:* theory-based determinants of intention (attitudes, subjective norms, perceived behavioural control and self-efficacy) and self-rated competence.

**Results:**

20 participants (6 female, 14 male), aged 20–84 provided baseline data. 17 received the full suite of 35 text messages.15 provided follow-up data. Intentions to perform CPR in scenarios where CPR was indicated were high at baseline and increased (18.1 ± 3.2–19.5 ± 1.8/21) after the intervention, as did self-efficacy and self-rated competency. Self-efficacy, attitudes, perceived behavioural control and subjective norms were positively correlated with intentions. Qualitative data suggest the intervention was perceived as useful. Additional options for delivery format and pace were suggested.

**Conclusions:**

Pilot-testing suggests a text-message intervention delivered after CPR training is acceptable and may be helpful in increasing/maintaining intentions to perform CPR.

## Introduction

Out of hospital cardiac arrest (OHCA) has very high mortality.^1^ In the UK, 10% of cases survive to hospital discharge^2^ whereas 25% is reported in the best performing European centres.^3^

Prompt, effective bystander cardiopulmonary resuscitation (CPR) is the most important factor determining survival from OHCA, increasing survival almost 4-fold.^4^, ^5^ Yet, up to a third of those who experience OHCA do not receive CPR prior to the arrival of emergency services.^6^ Training alone is not enough to ensure initiation of CPR– evidence shows that as few as half of CPR-trained individuals attempt CPR in OHCA.^7^ Means of increasing the *proportion* of trained lay-people who actually apply their skills in the event of OHCA are urgently required.^8^

The reasons people do not initiate CPR include knowledge deficits, social and structural barriers such as gender and socio-economic status^9^ but also behavioural and psychological factors. A recent systematic review found the overwhelming emotion of the situation, perceptions about capability, uncertainty about when CPR is appropriate, feeling unprepared and fear of doing harm to be particularly important [unpublished data]. Crucially, these factors are potentially amenable to change using behavioural science. Conceptualising CPR as behaviour and applying behavioural science offers a means to increase initiation.

Behavioural science is a discipline concerned with explaining and changing behaviour. Well-validated behavioural theories specify the main predictors of behaviour. The Theory of Planned Behaviour^10^ and Health Action Process Approach^11^ suggest that a necessary (but not sufficient) first step to undertaking any behaviour is for people to develop the ‘intention’ to perform that behaviour. Meta-analysis of a large body of evidence^12^ confirms three targets to increase intentions: (1) positive ***attitudes*** about the behaviour; (2) beliefs that others would approve of the behaviour (***perceived social norms***) and (3) beliefs about one’s capability to perform CPR (***perceived behavioural control).***

A taxonomy of behaviour-change techniques^13^ provides a range of options to change attitudes, norms and perceived behavioural control and thus intentions to perform CPR and importantly to support conversion of intentions into action (e.g. making plans to overcome potential barriers).

The aims of the study were (1) to pilot a developed intervention and explore participant responses to it and (2) to pilot measures for use in a future evaluation of effectiveness.

## Methods

This was a prospective, observational before and after pilot study plus qualitative interviews. Ethical approval was provided by University of Stirling General University Ethics Panel, Ref: GUEP 791.

### Participants

Members of the general public (*n* = 20) undertaking CPR training with a CPR training organisation. (e.g. Heartstart UK) or who had undertaken CPR training in the previous 2 years. This number was considered adequate to achieve a diverse range of participants and to purposively sample for qualitative interviews. We included adults aged over 18 years who were able to communicate in English, had access to a mobile telephone and text-messaging. All participants provided written informed consent.

### Recruitment

Participants were recruited in 3 ways.

1. Via an ‘Invitation to participate’ flyer sent by CPR training organisations to potential participants on behalf of the study team
2. Via social media (Twitter and Facebook)
3. In person at CPR training sessions (with permission from the organiser)

Interested individuals were provided information about the study and contact details recorded. The intention was to purposively select participants to achieve diversity in terms of age, gender, ethnicity, socio-economic group and geographical location but ultimately all who responded were included. Consenting participants provided their mobile telephone number and agreed to receive text messages over a 4–6 week period.

### The Intervention

We developed a text-message intervention Behavioural Intervention to Increase CPR Performance (BICeP) to deliver behaviour-change techniques as a supplement to ‘standard’ CPR training. A text-message mode of delivery was selected to: (1) facilitate engagement with trainees beyond initial training. (2) reinforce key messages, possibly extending the longevity of competence^14^ (3) be an engaging means of delivering the intervention^15^, ^16^ (4) include visual media which may enhance effectiveness^17^ and (5) be scalable at relatively low cost.

Consistent with Medical Research Council Framework for developing and evaluating complex interventions,^18^ three sources of data were used to develop the BICeP intervention:

1. a systematic review of the literature identified the predictors of CPR initiation (unpublished data),
2. qualitative interviews with lay people (*n* = 10) explored their perceptions of what would be appealing, engaging and acceptable.
3. Iterative feedback from lay-people (*n* = 7) and CPR training experts (*n* = 18) via dedicated Microsoft Teams channels, email and in-person meetings throughout the development process refined the intervention.

The BICeP intervention is intended to increase lay-people’s intentions to perform CPR in the event of OHCA and specified in accordance with international best practice for intervention description^19^, ^20^ (see supplemental file 2). BICeP comprises a series of 35 text-messages containing at least 14 behaviour-change techniques (verified by expert reviewers external to the project and listed in Box 1, below).

Box 1 **Behaviour change techniques included in BICeP (see Taxonomy for full definitions).13**

1.4 Action planning

1.9 Commitment

3.1 Social support (unspecified)

3.3 Social support (emotional)

4.1 Instruction on how to perform the behaviour

5.1 Information about health consequences

5.5 Anticipated regret

5.6 Information about emotional consequences

8.1 Behavioural practice/rehearsal

9.1 Credible source

9.2 Pro’s & Con’s

9.3 Comparative imagining of future outcomes

11.2 Reduce negative emotions

15.1 Verbal persuasion about capability

**Fig. 1:**
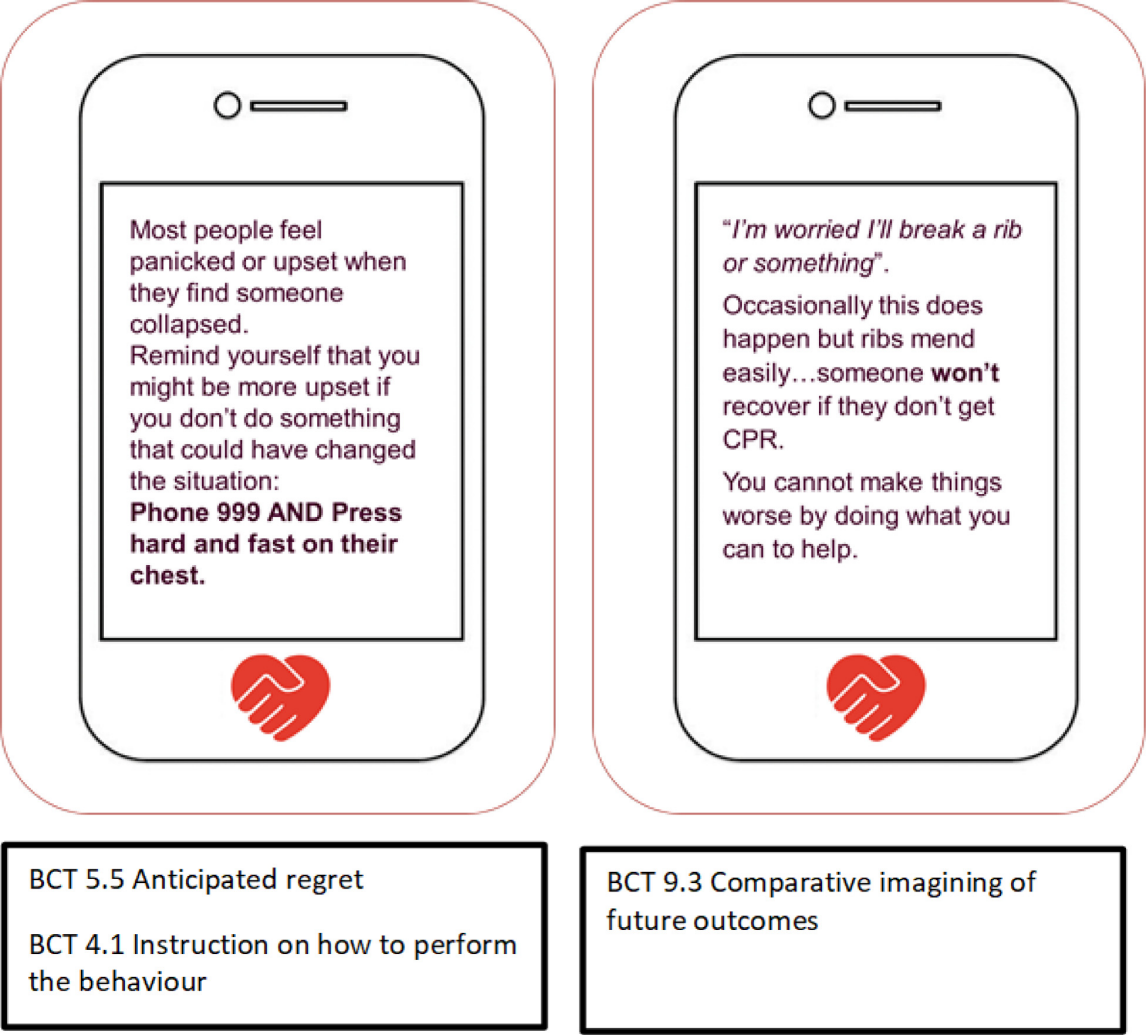
Example text messages.

Example messages are provided in Fig. 1.^1^ The order in which the messages were delivered was tailored based on participants’ level of intention (high or low) and their reported main concerns about doing CPR.

Twelve messages contained embedded links to CPR news articles or short videos (e.g. demonstrating agonal breathing). These were supplementary to the core intervention so that people who did not/were unable to view them still received, as a minimum, the 14 intended behaviour-change techniques. Fourteen messages had interactive elements, where participants were invited to carry out a personal activity (e.g. writing a plan to overcome a barrier) or reply to tailoring messages. Participants could reply to any text and request to stop messages at any time.

The 35 messages were designed to be delivered over 6 months, at a daily frequency for 2 weeks following initial training (to reinforce and consolidate its main messages and achieve engagement with the intervention), reducing to one message/week thereafter to avoid irritation^21^ However, for the purposes of the pilot study (the duration of which was time-restricted) messages were sent more frequently so participants could experience the entire intervention. Messages were sent every 1–2/days, usually at midday over a 4–6 week period.

### Data collection and analysis

Data collection took part between May and September 2021. Demographic data: gender, age, ethnicity, socioeconomic status, level of education, employment status, partial postcode and date of CPR training was recorded. Participants completed questionnaires (comprising the measures listed below) prior to taking part in the intervention (in-person) and 1 week after completion (by phone) (see supplemental file 1). Each participant was allocated a Study Identification (ID) number (known only to the research team) and a linked ID number for the automated text system. The researcher added the linked ID, participants’ first name and contact number to the automated text message system which triggered messages to begin on the following Monday.

#### Primary outcome measure: *Intention to initiate CPR*

Intentions to initiate CPR were assessed in response to 4 different scenarios using a single Likert-type item (“In this situation, I would begin CPR immediately”) scored 1 = strongly disagree to 7 = strongly agree. Three of the four scenarios were cardiac arrest scenarios. The intention score was calculated as the mean score across the three cardiac arrest scenarios.

Cognitive determinants of intention were assessed for each scenario, three items assessed *attitude* towards initiating CPR using semantic differential scales (e.g. For me to begin CPR immediately in this situation would be Useless-Useful) scored 1 = useless −7 = useful; three *subjective norm* items (e.g. people who are important to me would think I should perform CPR in this situation) scored 1 = strongly disagree to 7 = strongly agree; and three *perceived behavioural control* items (e.g. Whether or not I begin CPR in this situation is entirely up to me) scored 1 = strongly disagree to 7 = strongly agree. Mean for attitude, subjective norm and perceived behavioural control was calculated for each scenario and scores for the CPR scenarios only summed before and after intervention.

#### Self-efficacy

S*elf-efficacy* (or confidence) to perform CPR was assessed once before and once after the intervention. Participants rated how certain they were that they could perform CPR in ten different situations which varied in difficulty.

#### Self-evaluation of competence

Self-evaluated competence was assessed once before and after the intervention using an item previously published^22^. Participants were asked to respond to 4 statements indicating how able they felt to perform basic life support (e.g. I am completely unable) on a scale from 1 = strongly disagree to 7 = strongly agree.

#### Statistics

SPSS Version 28 (IBM, New York, USA) was used to calculate mean scores ± standard deviation for each measure were calculated before and after the intervention. This pilot study was designed to assess the acceptability of the intervention and the feasibility of a fully powered effectiveness trial, it was not powered for pre vs post-intervention comparisons, therefore, data are summarised using descriptive statistics. Pearson’s correlations were used to explore the relationship between theoretical predictors of intention and intention to initiate CPR, a *p* value of ≤0.05 being considered statistically significant.

#### Qualitative data

Ten participants were purposively selected (based on responses to the intervention [i.e. completion vs non-completion] and aiming for diversity) for individual interview. Within 4 weeks of end of intervention, participants were asked their views on the acceptability, usefulness and impact of the text-message intervention as well as the research methods used. Interviews were audio-recorded and transcribed. Transcripts were anonymised and transferred to NVivo 12 qualitative analysis software (QSR International UK Ltd.) for analysis.

Thematic analysis guided by the steps provided by Braun and Clarke^23^ was undertaken.

## Results

lay-people (6 female, 14 male) participated in the study (see Table 1). All 20 provided baseline data and 15 provided follow-up data after receipt of the intervention.

**Table 1:** Participant demographics compared to Scottish population.*

		Adult Population Scotland (%)	BiCEP Sample (%)
Gender	Female	51	30
	Male	49	70
Age	≤44 years old	43	60
	>44 years old	57	40
Education Level	School	64	10
	College	10	35
	Higher Ed+	26	55
Socio-economic status (Scottish Index of Multiple Deprivation)	1 (Most deprived) 2 3 4 5 (Least deprived)	20 20 20 20 20	0 20 25 50 5
Cohabiting	Married/cohabiting Live alone	20	75 25
Employment status	Employed Self-employed Retired		60 5 35
CPR training	First-time Refresher		50 50

* Age and gender based on National Records for Scotland mid-2020^25^; Education level and cohabitation from Census 2011.^26^

### Acceptability of the intervention

Messages were sent to 20 participants who received them over a period of 4–6 weeks. Three requests to STOP were received (15% of participants): one at message 1 (lost to follow-up); one after message 20 (family member became very ill) and one after message 22 (felt too busy at work). The remaining 17 participants received the full suite of messages (*n* = 35).

Fourteen messages invited a response (although it was not required). The rate of response varied by message from 0% (to a message inviting further question) to 60% (to a tailoring message differentiating high intentions from low). Response rates also varied between participants – three responded to none of the 14 messages, seven responded to four or more (maximum 9).

Qualitative interview data showed that participants considered most elements of the intervention useful and acceptable. The content of messages was generally considered appropriate and the intervention was praised.

“*It’s extremely worthwhile and if it’s making people feel a little bit more confident then that’s a good thing*.” (Participant 17: 65yrs, female, refresher training)

Daily frequency of the messages was acceptable for a short time but beyond the first few days, less frequent delivery would be preferred by participants. The brevity of the messages was reported as important, making it possible to read messages when busy. The mixture of styles, embedded short videos and news article links and interactive elements were well received.

Whilst most participants found the text format suitable, in areas with poor 4G, connecting to embedded videos or links was sometimes challenging. Some participants who did not use smart phone functionality regularly or who had ‘basic’ phones were less enthusiastic about text, some expressing a preference for emails.

### Impact of the intervention

**Fig. 2:**
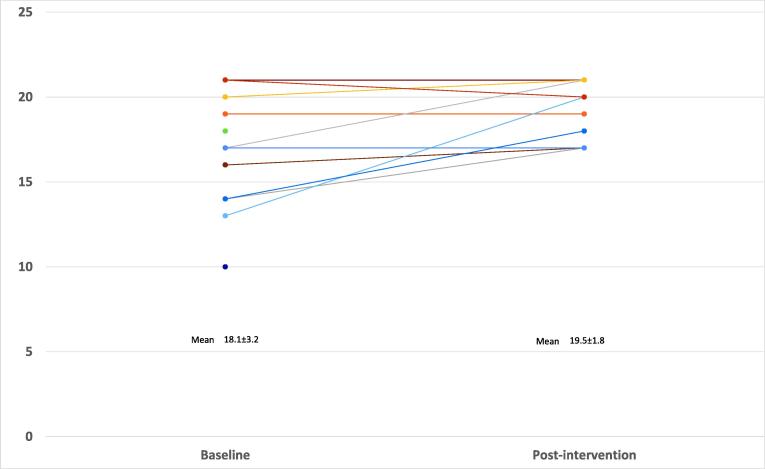
Primary outcome: Intention to perform CPR (before and after intervention).

Fig. 2 shows the intention at baseline and the intention post-intervention for each participant (data missing for 5 at follow-up). As can be seen in Fig. 2, most participants either maintained initial high intentions to perform CPR or increased intentions. Importantly, the largest increases were seen in those with the lowest initial intentions. Across participants, mean intentions increased from 18.1 ± 3.2 to 19.5 ± 1.8 (maximum possible = 21) following the intervention. Mean intentions in the *non-cardiac arrest* scenario were low at 2.2 ± 1.8 at baseline and 1.7 ± 1.5 following the intervention.

**Fig. 3:**
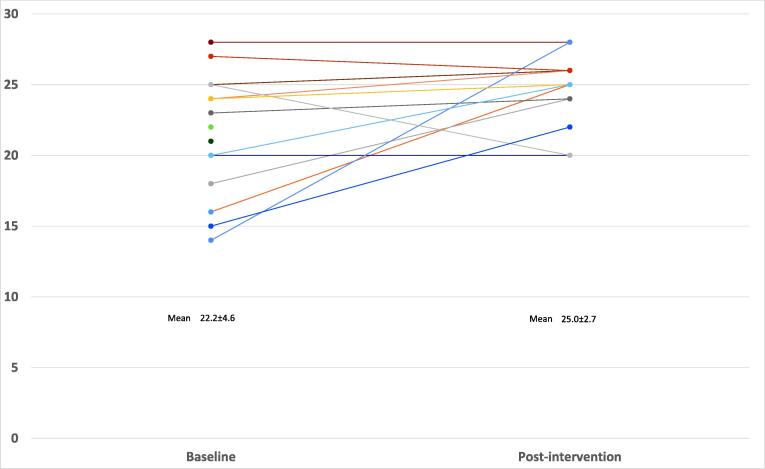
Self-rated competency (before and after intervention).

Fig. 3 shows similar improvements in self-rated competency: mean 22.2 ± 4.6 at baseline vs 25.0 ± 2.7 following the intervention (maximum score = 28). Again, the largest increases were seen in those with the lowest self-rated competency at baseline.

Theoretical predictors of intention (self-efficacy, attitudes, perceived behavioural control, subjective norms) also increased overall and were positively correlated with intention (see Table 2).

**Table 2:** Theoretical predictors of intention.

Theoretical Predictor (max possible score)	Baseline mean (SD)		Post-intervention mean (SD)		Correlation with intention (*r*)	*P*(sig.)
Attitudes (63)	56.2	(6.2)	59.9	(4.5)	0.94	<0.01
Perceived Social Norms (63)	51.7	(7.7)	54.5	(5.4)	0.67	<0.01
Perceived Behavioural Control (63)	50.1	(9.6)	56.3	(7.1)	0.67	<0.01
Self-efficacy (100)	73.0	(16.4)	78.0	(15.5)	0.63	=0.01

Qualitative data also suggest a positive impact of the intervention, particularly so for those who had undertaken CPR training some time ago.

*“If I was to look back on the day that they gave us the CPR training, I wouldn’t say that I do remember absolutely everything that they told us, so if I hadn’t got the intervention between now and when I had the training…I would say I would know a lot less than I do now from getting the texts every so often.”* (Participant 2: 28 yr, male, refresher training)

A key factor in increasing confidence was the regular reminder of the participant’s CPR training and of the importance of carrying out CPR when necessary.

“*I definitely think it increased my confidence by kind of feeling like, okay, these sort of again kind of refreshers or kind of bits of information that help remind me what to do in a situation and also I kind of have more confidence in the skills that I already know that I have, so kind of seeing those reminders helped to boost that.*” (Participant 7: 37 yr, female, first-time training)

This increase in confidence was perceived as having a potential positive impact on the likelihood of putting the training into practice if needed, and some participants were emphatic that the BiCEP intervention had increased their intention to carry out CPR. In some cases, proactive steps had been taken to prepare for the eventuality of performing CPR.

*“Had I not taken part in this I would’ve been confident to do the CPR but I wouldn’t have been confident enough to sort of lead a situation, like, manage a situation, whereas now I think I’d be quite forward with it, I’d be able to take control of the whole entire situation.” (Participant 2, 28 yr, male, refresher training)*

### Acceptability of study methods

Approach in person was preferred by participants and indeed the most successful method of recruitment. Being able to communicate with the research team directly reported as important in the decision to take part and building trust in the sender of the texts particularly important. Participants found the study methods generally acceptable but reported that four scenarios were too many, suggesting the number be reduced.

## Discussion

This study has shown that a theory-based text-messaging intervention designed to increase confidence and intentions to initiate CPR is acceptable to lay-people., Eighty-five percent of the participants in this pilot study accepted a condensed, intense version of the intervention over 4–6 weeks. Given interview participants specifically suggested reduced frequency of messages, a less intense version delivered over 6 months would be expected to be no less acceptable although how well engagement would be maintained over a longer period is unknown.

The study was not powered to support formal, statistical pre vs post-intervention comparisons. Descriptive analysis shows intentions, self-efficacy (confidence) and self-rated competence in relation to CPR scenarios were scored higher after the intervention than before, particularly for individuals who had lower intentions and self-rated competence at baseline. This suggests the intervention may have a positive impact and be a useful adjunct to CPR training. It is well-documented that CPR skills and knowledge deteriorate quickly within months of training, even amongst medical staff^24^ and nurses.^25^ Confidence declines too – Fratta et al. (2020) found the percentage of people reporting being very confident halved in the year following training. In this context, maintaining initial high intentions and confidence constitutes success, particularly when ceiling effects are considered (people scoring maximum at baseline cannot be improved).

Positive correlations between the theoretical predictors of intention and measures of intention suggest the intervention is operating via the mechanisms intended. This highlights the usefulness of a theory-based approach and also means that future iterations can be adapted (e.g. in light of new CPR guidance), while preserving (or indeed enhancing) essential behaviour change content. The intervention is novel and, as it can be delivered largely automatically, could readily be delivered to large numbers of people at relatively low cost.

The research design was generally acceptable to participants, but we have identified opportunities to strengthen the study design and intervention (e.g. approaching people in person at CPR training is critical and will be planned into larger scale evaluation). The number of scenarios could be reduced to 3 with little impact on rigour whilst reducing participant burden.

### Limitations

This pilot study was not designed or powered to make inferences about effectiveness. There was no control group and scores obtained after the intervention were obtained only from those who participated in follow-up, which may introduce bias.

As CPR is not a readily observable behaviour, we were confined to the use of self-report measures in relation to hypothetical scenarios, which may not reflect participants’ responses in real-life. We mitigated against socially desirable responses by including a scenario where CPR was not indicated (a chest pain scenario where the patient was still breathing) and participants’ self-reported intentions to perform CPR in that scenario were very low suggesting they were engaged meaningfully in their responses to the scenarios.

Follow-up was limited to 6 weeks only and so longer-term follow-up is required to assess if results are maintained longer term.

The study was conducted whilst government restrictions on social mixing related to the Covid-19 pandemic were in place and community CPR training was not being provided. This meant our sample was obtained predominantly from commercial providers of CPR training: females, people of non-white ethnic origin and from the lowest socio-economic group were under-represented (although they were represented during intervention development) – further evaluation with a more diverse sample is required and being planned.

## Conclusion

This pilot study showed that a text-message intervention delivered after CPR training is acceptable and that a larger scale study is feasible. Full scale evaluation is required to establish if it is effective in maintaining high intentions to perform CPR.

## Conflict of Interest Statement

The authors declare no conflicts of interest.

## CRediT authorship contribution statement

**Barbara Farquharson:** Conceptualization, Methodology, Validation, Data curation, Supervision, Project administration, Funding acquisition. **Eileen Calveley:** Investigation, Data curation, Writing – review & editing. **Gareth Clegg:** Methodology, Writing – review & editing, Funding acquisition. **Brian Williams:** Conceptualization, Methodology, Writing – review & editing, Funding acquisition. **Pam Ramsay:** Methodology, Writing – review & editing, Funding acquisition. **Lisa Macinnes:** Methodology, Writing – review & editing, Funding acquisition. **Claire Torrens:** Investigation, Writing – review & editing. **Diane Dixon:** Conceptualization, Methodology, Writing – review & editing, Funding acquisition.

## Footnotes

1 Full copies of messages not provided to avoid control-group contamination prior to planned full-scale evaluation.
