# Supplementary material for: Increasing lay-people’s intentions to initiate CPR in out of hospital cardiac arrest: Results of a mixed-methods ‘before and after’ pilot study of a behavioural text-message intervention (BICeP)
Source: Resusc Plus. 2022 Oct 6;12:100312. doi: 10.1016/j.resplu.2022.100312 (PMC9551210; doi:10.1016/j.resplu.2022.100312)
Supplement: Supplementary file 1 [file mmc1.docx]

**BiCEP Pilot Study Questionnaire**

**1. Performing CPR**

A number of situations are described below that other people have told us made it more or less difficult for them to perform CPR. Please rate how confident you are that you **could** perform CPR immediately in each situation, by circling your answer.

| **[SELF-EFFICACY]** | Not at all confident (0) | Moderately confident (5) | Highly confident (10) |
| --- | --- | --- | --- |
| If someone told you it was the right thing to do | 0 1 2 3 4 5 6 7 8 9 10 | | |
| If the situation was dangerous for you | 0 1 2 3 4 5 6 7 8 9 10 | | |
| If the victim was someone you care about | 0 1 2 3 4 5 6 7 8 9 10 | | |
| If there was no-one else around | 0 1 2 3 4 5 6 7 8 9 10 | | |
| If you were distressed | 0 1 2 3 4 5 6 7 8 9 10 | | |
| If there was blood or vomit on the victim | 0 1 2 3 4 5 6 7 8 9 10 | | |
| If the victim was a stranger | 0 1 2 3 4 5 6 7 8 9 10 | | |
| If the victim was a child | 0 1 2 3 4 5 6 7 8 9 10 | | |
| If a 999-call handler was telling you exactly what to do | 0 1 2 3 4 5 6 7 8 9 10 | | |
| If you knew you were protected from any legal issues | 0 1 2 3 4 5 6 7 8 9 10 | | |

**2. Example Scenarios**

In the next section you will be asked to think carefully and imagine yourself in a series of situations. You will then be asked some questions about what you think about the situation and how you would act in that situation. Please think about each situation carefully and then answer the questions, by circling your response.

**Scenario 1: You are returning home after a day out with friends and one of them suddenly collapses to the ground, unconscious. They are not breathing.**

| **[INTENTION]** |
| --- |
| a) In this situation, I would begin CPR immediately. |
| \| Strongly Disagree \| 1 \| 2 \| 3 \| 4 \| 5 \| 6 \| 7 \| Strongly agree \| \| --- \| --- \| --- \| --- \| --- \| --- \| --- \| --- \| --- \| |
| **[ATTITUDES]** |
| b) For me to begin CPR immediately in this situation would be: |
| \| Useless \| 1 \| 2 \| 3 \| 4 \| 5 \| 6 \| 7 \| Useful \| \| --- \| --- \| --- \| --- \| --- \| --- \| --- \| --- \| --- \|  \| Foolish \| 1 \| 2 \| 3 \| 4 \| 5 \| 6 \| 7 \| Wise \| \| --- \| --- \| --- \| --- \| --- \| --- \| --- \| --- \| --- \| \| Inappropriate \| 1 \| 2 \| 3 \| 4 \| 5 \| 6 \| 7 \| Appropriate \| |
| **[PERCEIVED SOCIAL NORMS]** |
| c) People who are important to me (e.g. spouse) would think I should/should not begin CPR in this situation. |
| \| Should not \| 1 \| 2 \| 3 \| 4 \| 5 \| 6 \| 7 \| should \| \| --- \| --- \| --- \| --- \| --- \| --- \| --- \| --- \| --- \| |
| d) People who are important to me would approve/disapprove of me performing CPR in this situation. |
| \| Disapprove \| 1 \| 2 \| 3 \| 4 \| 5 \| 6 \| 7 \| Approve \| \| --- \| --- \| --- \| --- \| --- \| --- \| --- \| --- \| --- \| |
| e) Most people in this situation would begin CPR |
| \| Strongly disagree \| 1 \| 2 \| 3 \| 4 \| 5 \| 6 \| 7 \| Strongly agree \| \| --- \| --- \| --- \| --- \| --- \| --- \| --- \| --- \| --- \| |
| **[PERCEIVED BEHAVIOURAL CONTROL]** |
| f) Performing CPR in this situation is beyond my control |
| \| Strongly disagree \| 1 \| 2 \| 3 \| 4 \| 5 \| 6 \| 7 \| Strongly agree \| \| --- \| --- \| --- \| --- \| --- \| --- \| --- \| --- \| --- \| |
| g) Whether or not I begin CPR in this situation is entirely up to me. |
| \| Strongly disagree \| 1 \| 2 \| 3 \| 4 \| 5 \| 6 \| 7 \| Strongly agree \| \| --- \| --- \| --- \| --- \| --- \| --- \| --- \| --- \| --- \| |
| h) It would be difficult for me to begin CPR in this situation. |
| \| Strongly disagree \| 1 \| 2 \| 3 \| 4 \| 5 \| 6 \| 7 \| Strongly agree \| \| --- \| --- \| --- \| --- \| --- \| --- \| --- \| --- \| --- \| |

**Scenario 1: You are returning home after a day out with friends and one of them suddenly collapses to the ground, unconscious. They are not breathing.**

| **[OUTCOME EXPECTANCIES]** | Strongly disagree (1) | | Neither agree nor disagree (4) | | | Strongly agree (7) | |
| --- | --- | --- | --- | --- | --- | --- | --- |
| I might save the person’s life | 1 | 2 | 3 | 4 | 5 | 6 | 7 |
| I will feel good about having tried to help | 1 | 2 | 3 | 4 | 5 | 6 | 7 |
| I might cause harm to the person | 1 | 2 | 3 | 4 | 5 | 6 | 7 |
| I will catch a disease | 1 | 2 | 3 | 4 | 5 | 6 | 7 |
| The person might die anyway | 1 | 2 | 3 | 4 | 5 | 6 | 7 |
| People will feel proud of me for trying CPR | 1 | 2 | 3 | 4 | 5 | 6 | 7 |

**Scenario 2: You are in a large shopping mall and you have to go to the toilet. There you find a lifeless stranger lying on the floor. The person is not breathing and is unconscious.**

| **[INTENTION]** |
| --- |
| a) In this situation, I would begin CPR immediately. |
| \| Strongly Disagree \| 1 \| 2 \| 3 \| 4 \| 5 \| 6 \| 7 \| Strongly agree \| \| --- \| --- \| --- \| --- \| --- \| --- \| --- \| --- \| --- \| |
| **[ATTITUDES]** |
| b) For me to begin CPR immediately in this situation would be: |
| \| Useless \| 1 \| 2 \| 3 \| 4 \| 5 \| 6 \| 7 \| Useful \| \| --- \| --- \| --- \| --- \| --- \| --- \| --- \| --- \| --- \|  \| Foolish \| 1 \| 2 \| 3 \| 4 \| 5 \| 6 \| 7 \| Wise \| \| --- \| --- \| --- \| --- \| --- \| --- \| --- \| --- \| --- \| \| Inappropriate \| 1 \| 2 \| 3 \| 4 \| 5 \| 6 \| 7 \| Appropriate \| |
| **[PERCEIVED SOCIAL NORMS]** |
| c) People who are important to me (e.g. spouse) would think I should/should not begin CPR in this situation. |
| \| Should not \| 1 \| 2 \| 3 \| 4 \| 5 \| 6 \| 7 \| should \| \| --- \| --- \| --- \| --- \| --- \| --- \| --- \| --- \| --- \| |
| d) People who are important to me would approve/disapprove of me performing CPR in this situation. |
| \| Disapprove \| 1 \| 2 \| 3 \| 4 \| 5 \| 6 \| 7 \| Approve \| \| --- \| --- \| --- \| --- \| --- \| --- \| --- \| --- \| --- \| |
| e) Most people in this situation would begin CPR |
| \| Strongly disagree \| 1 \| 2 \| 3 \| 4 \| 5 \| 6 \| 7 \| Strongly agree \| \| --- \| --- \| --- \| --- \| --- \| --- \| --- \| --- \| --- \| |
| **[PERCEIVED BEHAVIOURAL CONTROL]** |
| f) Performing CPR in this situation is beyond my control |
| \| Strongly disagree \| 1 \| 2 \| 3 \| 4 \| 5 \| 6 \| 7 \| Strongly agree \| \| --- \| --- \| --- \| --- \| --- \| --- \| --- \| --- \| --- \| |
| g) Whether or not I begin CPR in this situation is entirely up to me. |
| \| Strongly disagree \| 1 \| 2 \| 3 \| 4 \| 5 \| 6 \| 7 \| Strongly agree \| \| --- \| --- \| --- \| --- \| --- \| --- \| --- \| --- \| --- \| |
| h) It would be difficult for me to begin CPR in this situation. |
| \| Strongly disagree \| 1 \| 2 \| 3 \| 4 \| 5 \| 6 \| 7 \| Strongly agree \| \| --- \| --- \| --- \| --- \| --- \| --- \| --- \| --- \| --- \| |

**Scenario 2: You are in a large shopping mall and you have to go to the toilet. There you find a lifeless stranger lying on the floor. The person is not breathing and is unconscious.**

**If I perform CPR in this situation:**

| **[OUTCOME EXPECTANCIES]** | Strongly disagree (1) | | Neither agree nor disagree (4) | | | Strongly agree (7) | |
| --- | --- | --- | --- | --- | --- | --- | --- |
| I might save the person’s life | 1 | 2 | 3 | 4 | 5 | 6 | 7 |
| I will feel good about having tried to help | 1 | 2 | 3 | 4 | 5 | 6 | 7 |
| I might cause harm to the person | 1 | 2 | 3 | 4 | 5 | 6 | 7 |
| I will catch a disease | 1 | 2 | 3 | 4 | 5 | 6 | 7 |
| The person might die anyway | 1 | 2 | 3 | 4 | 5 | 6 | 7 |
| People will feel proud of me for trying CPR | 1 | 2 | 3 | 4 | 5 | 6 | 7 |

**Scenario 3: You are in the local newsagents when the shopkeepers suddenly exclaims in pain and grabs his chest. He looks very pale and tells you he feels sick.**

| **[INTENTION]** |
| --- |
| a) In this situation, I would begin CPR immediately. |
| \| Strongly Disagree \| 1 \| 2 \| 3 \| 4 \| 5 \| 6 \| 7 \| Strongly agree \| \| --- \| --- \| --- \| --- \| --- \| --- \| --- \| --- \| --- \| |
| **[ATTITUDES]** |
| b) For me to begin CPR immediately in this situation would be: |
| \| Useless \| 1 \| 2 \| 3 \| 4 \| 5 \| 6 \| 7 \| Useful \| \| --- \| --- \| --- \| --- \| --- \| --- \| --- \| --- \| --- \|  \| Foolish \| 1 \| 2 \| 3 \| 4 \| 5 \| 6 \| 7 \| Wise \| \| --- \| --- \| --- \| --- \| --- \| --- \| --- \| --- \| --- \| \| Inappropriate \| 1 \| 2 \| 3 \| 4 \| 5 \| 6 \| 7 \| Appropriate \| |
| **[PERCEIVED SOCIAL NORMS]** |
| c) People who are important to me (e.g. spouse) would think I should/should not begin CPR in this situation. |
| \| Should not \| 1 \| 2 \| 3 \| 4 \| 5 \| 6 \| 7 \| should \| \| --- \| --- \| --- \| --- \| --- \| --- \| --- \| --- \| --- \| |
| d) People who are important to me would approve/disapprove of me performing CPR in this situation. |
| \| Disapprove \| 1 \| 2 \| 3 \| 4 \| 5 \| 6 \| 7 \| Approve \| \| --- \| --- \| --- \| --- \| --- \| --- \| --- \| --- \| --- \| |
| e) Most people in this situation would begin CPR |
| \| Strongly disagree \| 1 \| 2 \| 3 \| 4 \| 5 \| 6 \| 7 \| Strongly agree \| \| --- \| --- \| --- \| --- \| --- \| --- \| --- \| --- \| --- \| |
| **[PERCEIVED BEHAVIOURAL CONTROL]** |
| f) Performing CPR in this situation is beyond my control |
| \| Strongly disagree \| 1 \| 2 \| 3 \| 4 \| 5 \| 6 \| 7 \| Strongly agree \| \| --- \| --- \| --- \| --- \| --- \| --- \| --- \| --- \| --- \| |
| g) Whether or not I begin CPR in this situation is entirely up to me. |
| \| Strongly disagree \| 1 \| 2 \| 3 \| 4 \| 5 \| 6 \| 7 \| Strongly agree \| \| --- \| --- \| --- \| --- \| --- \| --- \| --- \| --- \| --- \| |
| h) It would be difficult for me to begin CPR in this situation. |
| \| Strongly disagree \| 1 \| 2 \| 3 \| 4 \| 5 \| 6 \| 7 \| Strongly agree \| \| --- \| --- \| --- \| --- \| --- \| --- \| --- \| --- \| --- \| |

**Scenario 3: You are in the local newsagents when the shopkeepers suddenly exclaims in pain and grabs his chest. He looks very pale and tells you he feels sick.**

If I perform CPR in this situation:

| **[OUTCOME EXPECTANCIES]** | Strongly disagree (1) | | Neither agree nor disagree (4) | | | Strongly agree (7) | |
| --- | --- | --- | --- | --- | --- | --- | --- |
| I might save the person’s life | 1 | 2 | 3 | 4 | 5 | 6 | 7 |
| I will feel good about having tried to help | 1 | 2 | 3 | 4 | 5 | 6 | 7 |
| I might cause harm to the person | 1 | 2 | 3 | 4 | 5 | 6 | 7 |
| I will catch a disease | 1 | 2 | 3 | 4 | 5 | 6 | 7 |
| The person might die anyway | 1 | 2 | 3 | 4 | 5 | 6 | 7 |
| People will feel proud of me for trying CPR | 1 | 2 | 3 | 4 | 5 | 6 | 7 |

**Scenario 4: Your neighbour calls to say they aren’t feeling well and you pop round to check on them. When you enter the house, they are slumped in the chair, unconscious and not breathing.**

| **[INTENTION]** |
| --- |
| a) In this situation, I would begin CPR immediately. |
| \| Strongly Disagree \| 1 \| 2 \| 3 \| 4 \| 5 \| 6 \| 7 \| Strongly agree \| \| --- \| --- \| --- \| --- \| --- \| --- \| --- \| --- \| --- \| |
| **[ATTITUDES]** |
| b) For me to begin CPR immediately in this situation would be: |
| \| Useless \| 1 \| 2 \| 3 \| 4 \| 5 \| 6 \| 7 \| Useful \| \| --- \| --- \| --- \| --- \| --- \| --- \| --- \| --- \| --- \|  \| Foolish \| 1 \| 2 \| 3 \| 4 \| 5 \| 6 \| 7 \| Wise \| \| --- \| --- \| --- \| --- \| --- \| --- \| --- \| --- \| --- \| \| Inappropriate \| 1 \| 2 \| 3 \| 4 \| 5 \| 6 \| 7 \| Appropriate \| |
| **[PERCEIVED SOCIAL NORMS]** |
| c) People who are important to me (e.g. spouse) would think I should/should not begin CPR in this situation. |
| \| Should not \| 1 \| 2 \| 3 \| 4 \| 5 \| 6 \| 7 \| should \| \| --- \| --- \| --- \| --- \| --- \| --- \| --- \| --- \| --- \| |
| d) People who are important to me would approve/disapprove of me performing CPR in this situation. |
| \| Disapprove \| 1 \| 2 \| 3 \| 4 \| 5 \| 6 \| 7 \| Approve \| \| --- \| --- \| --- \| --- \| --- \| --- \| --- \| --- \| --- \| |
| e) Most people in this situation would begin CPR |
| \| Strongly disagree \| 1 \| 2 \| 3 \| 4 \| 5 \| 6 \| 7 \| Strongly agree \| \| --- \| --- \| --- \| --- \| --- \| --- \| --- \| --- \| --- \| |
| **[PERCEIVED BEHAVIOURAL CONTROL]** |
| f) Performing CPR in this situation is beyond my control |
| \| Strongly disagree \| 1 \| 2 \| 3 \| 4 \| 5 \| 6 \| 7 \| Strongly agree \| \| --- \| --- \| --- \| --- \| --- \| --- \| --- \| --- \| --- \| |
| g) Whether or not I begin CPR in this situation is entirely up to me. |
| \| Strongly disagree \| 1 \| 2 \| 3 \| 4 \| 5 \| 6 \| 7 \| Strongly agree \| \| --- \| --- \| --- \| --- \| --- \| --- \| --- \| --- \| --- \| |
| h) It would be difficult for me to begin CPR in this situation. |
| \| Strongly disagree \| 1 \| 2 \| 3 \| 4 \| 5 \| 6 \| 7 \| Strongly agree \| \| --- \| --- \| --- \| --- \| --- \| --- \| --- \| --- \| --- \| |

**Scenario 4: Your neighbour calls to say they aren’t feeling well and you pop round to check on them. When you enter the house, they are slumped in the chair, unconscious and not breathing.**

If I perform CPR in this situation:

| **[OUTCOME EXPECTANCIES]** | Strongly disagree (1) | | Neither agree nor disagree (4) | | | Strongly agree (7) | |
| --- | --- | --- | --- | --- | --- | --- | --- |
| I might save the person’s life | 1 | 2 | 3 | 4 | 5 | 6 | 7 |
| I will feel good about having tried to help | 1 | 2 | 3 | 4 | 5 | 6 | 7 |
| I might cause harm to the person | 1 | 2 | 3 | 4 | 5 | 6 | 7 |
| I will catch a disease | 1 | 2 | 3 | 4 | 5 | 6 | 7 |
| The person might die anyway | 1 | 2 | 3 | 4 | 5 | 6 | 7 |
| People will feel proud of me for trying CPR | 1 | 2 | 3 | 4 | 5 | 6 | 7 |

**3. To what extent do you feel able to perform basic life support?**

| **[SELF-ASESSED COMPETENCY]** | Strongly disagree (1) | | Neither agree nor disagree (4) | | | Strongly agree (7) | |
| --- | --- | --- | --- | --- | --- | --- | --- |
| I am completely unable | 1 | 2 | 3 | 4 | 5 | 6 | 7 |
| I am very uncertain and would probably not be able to help | 1 | 2 | 3 | 4 | 5 | 6 | 7 |
| I know the theory but not what to do in practice | 1 | 2 | 3 | 4 | 5 | 6 | 7 |
| I feel well prepared and will take action if a person falls over | 1 | 2 | 3 | 4 | 5 | 6 | 7 |
