# Supplementary material for: Increasing lay-people’s intentions to initiate CPR in out of hospital cardiac arrest: Results of a mixed-methods ‘before and after’ pilot study of a behavioural text-message intervention (BICeP)
Source: Resusc Plus. 2022 Oct 6;12:100312. doi: 10.1016/j.resplu.2022.100312 (PMC9551210; doi:10.1016/j.resplu.2022.100312)
Supplement: Supplementary file 2 [file mmc2.docx]

Table 1: TIDieR checklist[1]

|  | **TIDieR Description** | **Intervention Description** |
| --- | --- | --- |
| **BRIEF NAME** | Name or phrase that describes the intervention | A text-message behavioural Intervention to increase CPR Performance (BiCEP) |
| **WHY** | Describe any rationale, theory or goal of the elements essential to the intervention | To increase intention of lay-people to perform CPR in an out-of-hospital cardiac arrest (OHCA).  Based on a dual process model of behaviour change (HAPA) the messages comprise of information designed to create strong intentions to perform CPR, increase confidence in ability to perform CPR in a variety of circumstances and increase perceptions that doing CPR in the context of cardiac arrest is the right thing to do. It is intended to complement and augment initial CPR training of any format. |
| **WHAT** | Describe any physical or informational materials used in the intervention provided to participants or used in intervention delivery or in training of intervention providers. Provide information on where materials can be accessed.  Describe each of the procedures, activities, and/ or processes used  In the intervention including any enabling or support activities. | Materials  35 messages designed to be delivered by text-message to recipient’s mobile phones. Each message (except the initial introduction message) contains brief text containing at least one of the following BCTs:  1.4 Action planning  1.9 Commitment  3.1 Social support (unspecified)  3.3 Social support (emotional)  4.1 Instruction on how to perform the behaviour  5.1 Information about health consequences  5.5 Anticipated regret  5.6 Information about emotional consequences  8.1 Behavioural practice/rehearsal  9.1 Credible source  9.2 Pro’s & Con’s  9.3 Comparative imagining of future outcomes  11.2 Reduce negative emotions  15.1 Verbal persuasion about capability  Procedures  In some messages additional information, in the form of videos, online newspaper articles etc, is provided via hyperlink. The information is hosted on a dedicated web-page and participants access this by clicking on a hyper link in the text message.  The messages are available from the authors (not currently being publicly shared to avoid contamination of control group prior to main trial of effectiveness) |
| **WHO PROVIDED** | For each category of intervention provider (e.g. psychologist) describe their expertise, background and any specific training given. | Text messages were uploaded to an administrative system and delivered automatically to an agreed schedule by the Health Informatics Centre University of Dundee. The study team had administrative access. |
| **HOW** | Describe the modes of delivery (e.g. face-to-face, telephone) of the intervention and whether it was provided individuals or in a group. | Individuals were sent text messages to their mobile phone (number registered with the study). These were pre-scheduled. 14 messages invited a response but it was not required. At any time participants could reply with questions/comments and the research team alerted via a ‘dashboard’ |
| **WHERE** | Describe the type(s) of location(s) where the intervention occurred including any necessary infrastructure or relevant features. | The intervention was provided to individuals via mobile phone and they engaged with message content in the location they choose (either at time of receipt or later). |
| **WHEN and HOW MUCH** | Describe the number of times the intervention was delivered and over what period of time including number of sessions, their schedule, duration, intensity or dose. | Individuals received 35 text messages over 4-6 weeks. Messages were sent daily at 12MD for the first 13 days and then reduced to around every 2 days for the remainder. |
| **TAILORING** | If the intervention was planned to be personalised, titrated or adapted describe what, why, when, and how | Following the ‘welcome’ message, a tailoring message assesses intentions to initiate CPR on a scale of 1-5 (1-not at all likely – 5 very likely). Those who score ≤3 are categorised as low intention and those scoring 3+, high intention. Both groups receive a message of support but the wording differs – ‘You don’t sound as sure as we’d like’ for those with low intentions and ‘we’re glad you feel confident – we are going to send you messages to keep you feeling that way’ for those with high intentions. Both groups are invited to respond to the message with any areas that they would particularly want addressed and the subsequent ordering of messages is set to reflect this (with messages to address their priorities) sent first. Individuals’ names were used within the texts to personalise the message. |
| **MODIFICATIONS** | If the intervention was modified during the course of the study, describe the changes (what, why, when, and how) | No modifications were made during the pilot study |
| **HOW WELL** | Planned: If intervention adherence or fidelity was assessed, describe how and by whom and if any strategies were used to maintain or improve fidelity, describe them.  Actual: If intervention adherence or fidelity was assessed, describe the extent to which the intervention was delivered as planned. | Ten participants who participated in a qualitative interviews confirmed receipt of all the messages with the exception of message 10 (which contained a picture) |

1. Hoffmann, T.C., et al., *Better reporting of interventions: template for intervention description and replication (TIDieR) checklist and guide.* BMJ, 2014. **348**.
